# Ureteric stricture: an unusual presentation of metastatic prostate adenocarcinoma

**DOI:** 10.1308/003588412X13373405385971

**Published:** 2012-10

**Authors:** S Jallad, R Turo, M Kimuli, J Smith, S Jain

**Affiliations:** Leeds Teaching Hospitals NHS Trust,UK

**Keywords:** Prostate, Prostate cancer, Prostate adenocarcinoma, Ureteric stricture

## Abstract

We describe an unusual case of a prostatic adenocarcinoma presenting with a ureteric stricture secondary to a discrete metastatic lesion. A 76-year-old man presented with a short history of right loin pain. Initial examination was unremarkable, digital rectal examination was normal and prostate specific antigen was within normal range. Computed tomography showed right hydronephrosis and a distal ureteric stricture. A distal ureteric transitional cell carcinoma was thought to be most likely. A nephroureterectomy was carried out and histology revealed a skipped lesion of a metastatic prostate adenocarcinoma. Metastatic lesions to the ureters due to prostate cancer are rare. It was believed to be secondary to a transitional cell carcinoma as there was no evidence initially to suggest prostatic disease as the cause. A prostatic adenocarcinoma should be considered in the differential diagnosis of any lesions in the ureter believed to have a malignant origin.

Ureters are often affected in prostate cancer, either by local extension causing obstruction or by external compression secondary to enlarged lymph nodes. Clinical presentation with ureteric involvement due to a discrete metastatic lesion is rare. We report an unusual case of prostate cancer presenting as a skipped lesion to the ureter.

## Case history

A 67-year-old man presented with a short history of right loin pain. Abdominal examination was unremarkable and digital rectal examination (DRE) revealed a smooth, benign feeling prostate with some firmness noted at the base. His creatinine was elevated to 193μmol/l (normal range: 80–115μmol/l), while the prostate specific antigen (PSA) level was 3.9ng/l and the liver function tests were within normal range. Subsequent computed tomography showed a right distal ureteric stricture causing right hydronephrosis and hydroureter. In addition, there was peristricture inflammatory changes and local lymphadenopathy.

The patient was discussed at a local multidisciplinary team meeting where it was thought that a distal ureteric transitional cell carcinoma (TCC) was the most likely diagnosis but that an inflammatory process was also a possibility. He was booked for an urgent right retrograde study and a right ureteroscopy.

Cystoscopy was normal. The right retrograde study showed a very tight long ureteric stricture, 4cm from the vesicoureteric junction, that was too tight to allow for ureteroscopy. A ureteric stent was therefore inserted and dimercaptosuccinic acid (DMSA) renography was arranged to assess the function of that kidney.

**Figure 1:**
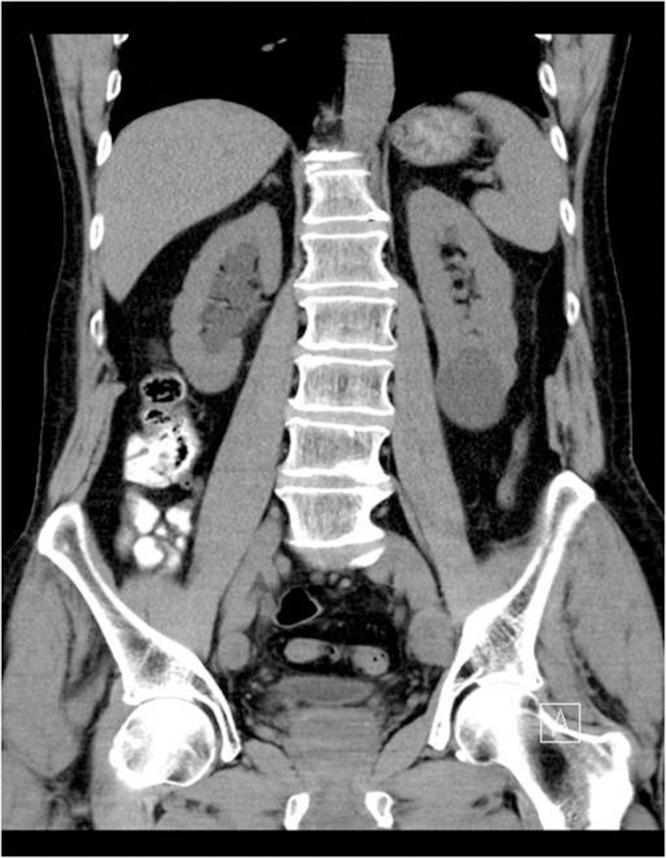
Computed tomography showing right hydronephrosis

**Figure 2:**
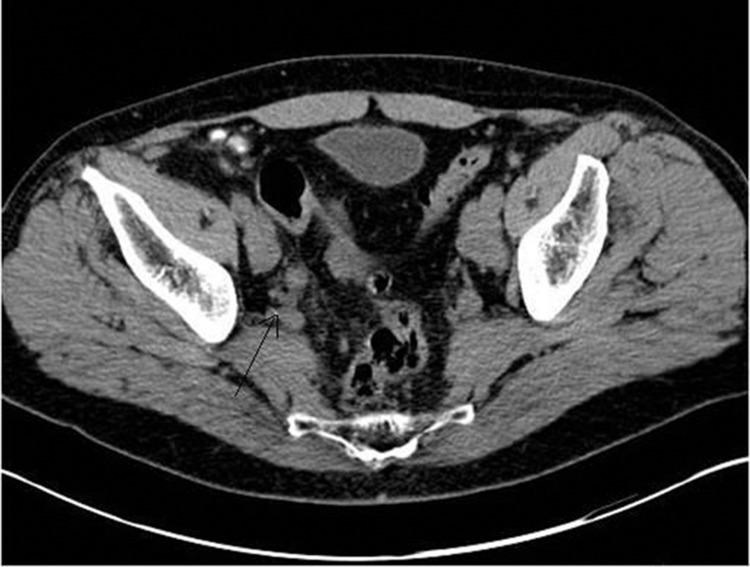
Computed tomography showing right ureteric thickening and local lymphadenopathy

**Figure 3:**
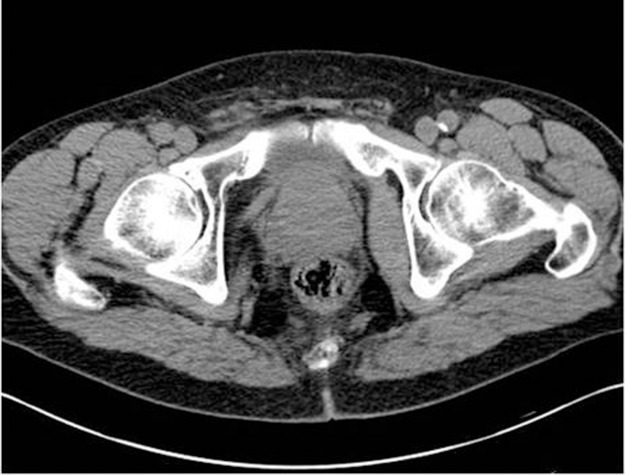
Computed tomography showing no advanced prostate disease and no metastatic bony disease

The DMSA showed a relative renal function of 20% on the right side. In view of poor renal function, the surgeon proceeded to open exploration and frozen section. The lower ureter was found to be very thickened and adherent to the pelvic sidewall with surrounding enlarged lymph nodes. Frozen section of the lymph nodes confirmed a carcinoma and a right nephroureterectomy was carried out.

The histology results showed a skipped lesion of a metastatic prostatic adenocarcinoma (Gleason grade 5+5) with positive lymph node metastasis and no evidence of TCC. The distal ureteric surgical margin was well clear of tumour.

The patient was seen four weeks later in the clinic and re-assessed. His DRE revealed a large irregular hard prostate. His PSA was found to be 101ng/l and his alkaline phosphatase had risen to 1,402iu/l (normal range: 70–300iu/l). His bone scan revealed widespread bony metastatic disease and he was commenced on gonadotropin releasing hormone analogue injection to treat the metastatic prostatic adenocarcinoma.

## Discussion

Prostatic adenocarcinoma presentation with a ureteric stricture secondary to a discrete metastatic lesion and a normal initial DRE and PSA is rare and has not been reported previously. To our knowledge, this is the first case in the literature of prostate cancer presenting with an isolated ureteric stricture.

We performed a MEDLINE® search and found 11 cases in 8 reports of metastatic lesions to the ureter.^1–8^ In the French literature, Petit *et al* reported two cases^1^ and Yonneau *et al* reported one case.^2^ Cortadellas *et al* reported one case in the Spanish literature.^3^ Brotherus and Westerlund reported three cases.^4^ Zollinger *et al*,^5^ Benejam *et al*,^6^ and Hulse and O’Neill^7^ reported one case each. Haddad reported three cases, one of which was caused by prostate cancer.^8^ To our knowledge, all these reports were in patients who had established prostate cancer or who had previously had treatment for prostate cancer. We also found reports of direct extension of prostate cancer involving one of the two ureters.^9,10^

## Conclusions

Our case represents an unusual first presentation of prostate cancer with initial normal examination and DRE. Metastatic lesions to the ureters due to prostate cancer are rare. A first presentation with this is unusual. Due to the presence of lymphadenopathy, a cancerous cause was suspected. It was believed to be secondary to TCC as there was no evidence initially to suggest prostatic disease as the cause. Prostatic adenocarcinoma should be considered in the differential diagnosis of any lesions in the ureter believed to have a malignant origin.

## References

[1] Petit J, Lesueur P, Petit J, Abourachid H. Ureteral metastases from prostatic cancers. 2 cases. Review of the literature. J Urol Nephrol (Paris) 1978; 84: 705–713 745253

[2] Yonneau L, Lebret T, Hervé JM*et al* Isolated ureteral metastasis of prostatic adenocarcinoma. Apropos of a case. Prog Urol 1999; 9: 118–121 10212962

[3] Cortadellas R, Prieto V, Castellanos R*et al* Ureteral metastasis of prostatic adenocarcinoma. Review of the literature. Arch Esp Urol 1989; 42: 19–22 2653236

[4] Brotherus JV, Westerlund RM. Metastatic carcinoma of the ureter. A report of three cases. Scand J Urol Nephrol 1971; 5: 86–90 5093100 10.3109/00365597109133583

[5] Zollinger RW, Wise HA, Clausen KP. An unusual presentation of intrinsic ureteral obstruction secondary to carcinoma of the prostate: a case report. J Urol 1981; 125: 132–133 7463572 10.1016/s0022-5347(17)54931-9

[6] Benejam R, Carroll TJ, Loening S. Prostate carcinoma metastatic to ureter. Urology 1987; 29: 325–327 3824736 10.1016/0090-4295(87)90084-7

[7] Hulse CA, O’Neill TK. Adenocarcinoma of the prostate metastatic to the ureter with an associated ureteral stone. J Urol 1989; 142: 1,312–1,313 2810518 10.1016/s0022-5347(17)39070-5

[8] Haddad FS. Metastases to the ureter. Review of the world literature, and three new case reports. J Med Liban 1999; 47: 265–271 10641458

[9] Chalasani V, Macek P, O’Neill GF, Barret W. Ureteric stricture secondary to unusual extension of prostatic adenocarcinoma. Can J Urol 2010; 17: 5,031–5,034 20156388

[10] Matsuo T, Hayashida Y, Takehara K*et al* A case of prostate cancer with invasion to the middle ureter. Hinyokika Kiyo 2004; 50: 61–63 15032020

